# Opioid-Induced Pruritus: A Case Series on Novel Uses of Nalbuphine in the Emergency Department

**DOI:** 10.1016/j.acepjo.2025.100266

**Published:** 2025-11-01

**Authors:** Aurelio A. Muzaurieta, Yoseph Z. Semma, Christopher C. Fernandes, Shawna Greenleaf, Jonathan B. Lee

**Affiliations:** 1 Department of Emergency Medicine, Stanford University, Palo Alto, California, USA; 2 Department of Anesthesiology, Perioperative, and Pain Medicine, Stanford University, Stanford, California, USA

**Keywords:** pain management, opioid, opioid-induced pruritus, nalbuphine

## Abstract

Opioid-induced pruritus is a frequent and distressing adverse effect of mu-opioid receptor agonists, often unresponsive to antihistamines such as diphenhydramine. Nalbuphine, a mixed kappa-opioid receptor agonist and mu-opioid receptor antagonist, offers both analgesia and pruritus prevention via targeted receptor mechanisms. We present 2 cases highlighting nalbuphine’s utility in the emergency department: one demonstrating prophylactic low-dose nalbuphine (2.5 mg IV) coadministered with hydromorphone in a pruritus-prone patient and one where nalbuphine (10 mg IV) served as a primary analgesic. In both cases, patients achieved pain relief without experiencing pruritus or sedation. These cases underscore nalbuphine’s potential as a safe and evidence-informed alternative in acute care settings.

## 1 Introduction

Nalbuphine is a synthetic opioid with a unique pharmacologic profile: a kappa-opioid receptor (KOR) agonist and a mu-opioid receptor (MOR) antagonist. This dual action provides analgesia while minimizing adverse effects associated with MOR agonists, including opioid-induced pruritus (OIP), respiratory depression, and nausea.^1^, ^2^, ^3^ First approved as an analgesic in 1979, nalbuphine is now generic and Food and Drug Administration–approved for the management of moderate to severe pain. It is administered intravenously (IV), intramuscularly (IM), or subcutaneously. Nalbuphine has been used off-label in numerous settings for both prevention and treatment of OIP.^2^

Nalbuphine’s kappa agonism induces analgesia through spinal cord and supraspinal pathways, whereas its mu antagonism attenuates MOR-mediated adverse effects without reversing analgesia.^1^^,^^2^ The activation of KORs is hypothesized to reduce the release of neurotransmitters involved in pain signaling.^1^^,^^4^ This receptor distribution and function supports nalbuphine’s role in providing effective analgesia with fewer adverse effects. Recommended dosing for analgesia is typically 10 mg IV/IM every 3-6 hours (max single dose: 20 mg), with a ceiling effect seen near 30 mg.^3^ For OIP, effective doses range from 2.5 to 5 mg IV for treatment and from 0.1 to 0.2 mg/kg IV for prophylaxis.^2^

Much of the literature supporting nalbuphine’s use in OIP comes from perioperative settings, particularly following intrathecal or epidural morphine, where it has proved efficacious in both prophylaxis and treatment of pruritus. Randomized controlled trials have shown its effectiveness for both prevention and treatment of OIP, without compromising analgesia or increasing sedation when used at antipruritic doses.^2^ A 2016 systematic review found that nalbuphine significantly reduced the incidence of neuraxial OIP compared with placebo or other agents and recommended its use as a first-line treatment in this setting.^2^ There is growing support for nalbuphine’s efficacy in OIP from parenteral or patient-controlled analgesia (PCA) opioid administration. Studies have demonstrated that combining nalbuphine with morphine in PCA provides additive analgesia, ratio-dependent antipruritic effects, and reduces opioid-related nausea without increasing opioid requirements.^5^^,^^6^ In addition to its benefit in prophylaxis and treatment of OIP, a meta-analysis of randomized controlled trials supports nalbuphine’s comparable analgesic efficacy to morphine but with fewer side effects.^7^

Understanding nalbuphine’s clinical role requires a nuanced appreciation of the pathophysiology of OIP. OIP is a complex phenomenon involving both central and peripheral mechanisms. Centrally mediated OIP results due to MOR activation in the spinal cord, triggering itch pathways independent of histamine release.^8^, ^9^, ^10^ This central mechanism helps explain why antihistamines like diphenhydramine have limited efficacy in OIP; their primary effect is sedation, which may interrupt the scratch-itch cycle rather than provide true antipruritic relief.^11^ Peripheral mechanisms, including nonimmunologic mast cell degranulation and pseudoallergic responses, can also contribute to pruritus via histamine release. However, these are less common and typically present with additional features such as flushing, urticaria, or hypotension. The majority of clinically significant OIP, especially following intravenous or neuraxial opioid administration, results from central MOR activation rather than peripheral histamine-mediated pathways.^8^^,^^9^^,^^10^

Given its mechanism of action, nalbuphine may be a promising pharmacologic option for managing both pain and pruritus in acute care settings. Its safety profile is superior to that of morphine with respect to pruritus and respiratory depression, as demonstrated in meta-analyses and systematic reviews.^2^^,^^7^ These properties make nalbuphine especially suitable for patients at risk of OIP or respiratory depression and further support its underexplored potential in the emergency department—particularly as an alternative to diphenhydramine and traditional opioids in pruritus-prone patients.

## 2 Case 1

A 51-year-old woman with a history of recurrent pancreatitis and ulcerative colitis status post proctocolectomy and prior small bowel obstructions (SBO) presented to the emergency department (ED) with acute abdominal pain, nausea, and vomiting. She was hemodynamically stable, and laboratory studies revealed an elevated white blood cell count that raised concern for an acute inflammatory or infectious process. Electrolytes, lipase, renal, and hepatic function were within normal limits. A computered tomography of the abdomen and pelvis revealed a high-grade SBO at the site of a previous anastomosis.

Given her pain severity, opioid therapy was indicated. However, the patient had a history of significant OIP without rash or other signs of allergic response, suggesting a centrally mediated mechanism. Chart review confirmed previous coadministrations of diphenhydramine with hydromorphone during previous hospitalizations to prevent itching.

To mitigate this adverse effect, the patient was coadministered 2.5 mg IV nalbuphine and 0.5 mg IV hydromorphone. This combination provided effective analgesia without inducing pruritus. The patient was admitted to general surgery, and nalbuphine 2.5 mg IV every 4 hours as needed for pruritus was administered with hydromorphone.

On hospital day (HD) 2, the patient underwent exploratory laparotomy with lysis of adhesions, resection of the obstructed anastomosis, and creation of a new anastomosis. In the postanesthesia care unit, a PCA with hydromorphone was initiated and nalbuphine for pruritus prophylaxis was continued. The nalbuphine dose was increased to 5 mg IV every 3 hours while on PCA, resulting in adequate pain relief without any side effects.

Nalbuphine was continued throughout her hospitalization with scheduled or as-needed doses coadministered with opioids. She tolerated the regimen well, experienced no pruritus or other side effects, and did not require antihistamines. She was discharged on HD 10 in improved condition.

## 3 Case 2

A 63-year-old man with a history of coronary artery disease status post drug-eluting stent to the distal right coronary artery, prior cerebrovascular accident, type 2 diabetes mellitus, obstructive sleep apnea, gout, and provoked pulmonary emboli on apixaban presented to the ED with atraumatic right knee pain and lower leg swelling for 4 days. He was afebrile and hemodynamically stable.

Physical examination revealed pain with active range of motion of the right knee but no joint effusion, warmth, or erythema. Knee tenderness was present, but there was no calf tenderness. Peripheral pulses were normal. Laboratory tests, radiographs, and venous ultrasound were unremarkable. X-ray imaging showed mild degenerative changes without acute fracture or dislocation. The findings were most consistent with a musculoskeletal source of pain, possibly degenerative or gout-related.

Given the patient’s documented history of OIP with hydromorphone, nalbuphine was initiated for analgesia. He received 10 mg of IV nalbuphine, resulting in significant pain reduction and improved knee mobility. He successfully ambulated in the ED without pruritus, sedation, or other adverse effects. He expressed satisfaction with treatment and inquired about outpatient nalbuphine, but was informed that no oral formulation is currently available. Given his history of gout, he was discharged with a short course of prednisone.

Three weeks later, the patient returned to the ED with 6 days of worsening bilateral knee and left ankle pain. He reported functional decline requiring assistance from 2 people to reach the hospital from his car. Evaluation was most consistent with a polyarticular gout flare refractory to colchicine and corticosteroids. He was admitted for pain management and further treatment of refractory gout. Nalbuphine 10 mg IV was again administered for analgesia with excellent effect and no recurrence of pruritus or sedation. His flare was managed with high-dose allopurinol and anakinra, and nalbuphine was continued throughout his hospitalization. He was discharged on HD 7 in improved condition.

## 4 Discussion

Nalbuphine’s pharmacologic properties make it uniquely suited for managing opioid-induced side effects, particularly pruritus. Its action as a MOR antagonist counters the central mechanism of OIP, whereas its kappa agonism provides analgesia with less risk of respiratory depression or euphoria.^1^^,^^2^ The ceiling effect of nalbuphine—approximately 30 mg—may limit its role in escalating or severe pain, but it is ideal in acute care settings where controlling moderate pain and mitigating opioid side effects are primary goals. Nalbuphine also demonstrates a ceiling effect for respiratory depression, which further supports its favorable safety profile.^12^

Diphenhydramine—though commonly used off-label—has limited effectiveness in histamine-independent, centrally mediated OIP, which is the predominant mechanism in most cases. Its perceived benefit is often due to sedation rather than a true antipruritic effect, and it carries risks of anticholinergic side effects such as delirium, cognitive impairment, and urinary retention, especially in older adults.^8^ Furthermore, antihistamines have been known to be misused in combination with opioids to enhance euphoria, a practice associated with significant health risks, including excessive sedation, potentially fatal respiratory depression, and potentiation of substance use disorder.^13^

Nalbuphine, by contrast, offers a mechanism-informed and repeatable approach to both pain and pruritus management. When used in conjunction with full agonists at lower doses to prevent itch, it does not compromise analgesic effects. It is well tolerated, does not cause significant sedation, and has a lower potential for abuse, making it an appealing option in patients with opioid sensitivity or at risk for misuse. Importantly, nalbuphine has been shown to produce naloxone-like effects without opioid agonist activity in methadone-dependent subjects, suggesting a potential to induce withdrawal.^14^ Therefore, we recommend continued monitoring and serial examination of chronic opioid-dependent patients, in whom opioid withdrawal can be precipitated.

Its underutilization in emergency medicine is likely multifactorial—stemming from limited ED-specific data, lack of formulary inclusion, and unfamiliarity among prescribers. These cases demonstrate that nalbuphine is a promising candidate for multimodal pain management in emergency settings. With appropriate clinical awareness, nalbuphine can fill a valuable niche as both a primary analgesic and adjunct in opioid-intolerant or pruritus-prone patients.

## 5 Conclusion

These 2 cases demonstrate the safe and effective use of nalbuphine in the ED, both as a prophylactic adjunct to prevent OIP and as a standalone analgesic. Nalbuphine’s dual receptor action provides targeted control of both pain and pruritus, reducing the need for sedating antihistamines and offering an alternative to conventional mu-agonist opioids. Its favorable safety profile and low abuse potential support further study and integration into emergency pain management protocols.

## Funding and Support

By *JACEP Open* policy, all authors are required to disclose any and all commercial, financial, and other relationships in any way related to the subject of this article as per ICMJE conflict of interest guidelines (see www.icmje.org). The authors have stated that no such relationships exist.

## Conflict of Interest

All authors have affirmed they have no conflicts of interest to declare.
